# Comparison of Head Impact Exposure Between Concussed Football Athletes and Matched Controls: Evidence for a Possible Second Mechanism of Sport-Related Concussion

**DOI:** 10.1007/s10439-018-02136-6

**Published:** 2018-10-22

**Authors:** Brian D. Stemper, Alok S. Shah, Jaroslaw Harezlak, Steven Rowson, Jason P. Mihalik, Stefan M. Duma, Larry D. Riggen, Alison Brooks, Kenneth L. Cameron, Darren Campbell, John P. DiFiori, Christopher C. Giza, Kevin M. Guskiewicz, Jonathan Jackson, Gerald T. McGinty, Steven J. Svoboda, Thomas W. McAllister, Steven P. Broglio, Michael McCrea, the CARE Consortium Investigators

**Affiliations:** 1 Joint Department of Biomedical Engineering, Marquette University and Medical College of Wisconsin, Milwaukee, WI USA; 2 Department of Neurosurgery, Medical College of Wisconsin, Milwaukee, WI USA; 3 Zablocki Veterans Affairs Medical Center, Milwaukee, WI USA; 4 Department of Epidemiology and Biostatistics, Indiana University School of Public Health, Bloomington, IN USA; 5 Department of Biomedical Engineering and Mechanics, Virginia Tech, Blacksburg, VA USA; 6 Departments of Neurosurgery and Pediatrics, UCLA Steve Tisch BrainSPORT Program, David Geffen School of Medicine, University of California, Los Angeles, Los Angeles, CA USA; 7 Division of Sports Medicine and Non-Operative Orthopaedics, Department of Family Medicine and Orthopaedics, David Geffen School of Medicine, University of California, Los Angeles, Los Angeles, CA USA; 8 Department of Orthopedics, School of Medicine and Public Health, University of Wisconsin, Madison, WI USA; 9 Matthew Gfeller Sport-Related Traumatic Brain Injury Center, University of North Carolina at Chapel Hill, Chapel Hill, NC USA; 10 John A. Feagin Jr. Sports Medicine Fellowship, Keller Army Hospital, United States Military Academy, West Point, NY USA; 11 Department of Sports Medicine, United States Air Force Academy, Colorado Springs, CO USA; 12 Department of Psychiatry, Indiana School of Medicine, Indianapolis, IN USA; 13 School of Kinesiology, University of Michigan, Ann Arbor, MI USA

**Keywords:** Repetitive head impact exposure, Subconcussive, Traumatic brain injury, Sport-related concussion

## Abstract

Studies of football athletes have implicated repetitive head impact exposure in the onset of cognitive and brain structural changes, even in the absence of diagnosed concussion. Those studies imply accumulating damage from successive head impacts reduces tolerance and increases risk for concussion. Support for this premise is that biomechanics of head impacts resulting in concussion are often not remarkable when compared to impacts sustained by athletes without diagnosed concussion. Accordingly, this analysis quantified repetitive head impact exposure in a cohort of 50 concussed NCAA Division I FBS college football athletes compared to controls that were matched for team and position group. The analysis quantified the number of head impacts and risk weighted exposure both on the day of injury and for the season to the date of injury. 43% of concussed athletes had the most severe head impact exposure on the day of injury compared to their matched control group and 46% of concussed athletes had the most severe head impact exposure for the season to the date of injury compared to their matched control group. When accounting for date of injury or season to date of injury, 72% of all concussed athletes had the most or second most severe head impact exposure compared to their matched control group. These trends associating cumulative head impact exposure with concussion onset were stronger for athletes that participated in a greater number of contact activities. For example, 77% of athletes that participated in ten or more days of contact activities had greater head impact exposure than their matched control group. This unique analysis provided further evidence for the role of repetitive head impact exposure as a predisposing factor for the onset of concussion. The clinical implication of these findings supports contemporary trends of limiting head impact exposure for college football athletes during practice activities in an effort to also reduce risk of concussive injury.

## Introduction

Sport-related concussion (SRC) has become increasingly recognized as a significant public health issue,^54^ known to produce acute changes in brain function resulting in an array of postconcussive symptoms and functional impairments. There is also increased concern about the effects of repetitive concussion, which may include persistent neuropsychiatric symptoms, cognitive dysfunction and neurodegenerative disease.^23^,^24^,^37^ It has long been understood that the biomechanical mechanism of concussion is associated with head impact resulting in linear and rotational accelerations.^27^,^39^ To investigate concussion biomechanics in a sports setting, electronic sensor systems were developed to quantify head impact accelerations. The most commonly used system is the Head Impact Telemetry (HIT) System, which consists of a series of accelerometers placed between the padding gaps of Riddell football helmets.^18^ The HIT System was used to quantify head impact biomechanics associated with concussion in football, resulting in the development of injury risk curves using regression techniques.^13^,^22^,^45^ The implicit assumption of these analyses is that the biomechanical onset of concussion (i.e., the concussive injury) was associated with a single head impact event. The single impact mechanism of concussion is supported by a wealth of experimental studies.^32^,^40^–^42^,^49^

More recently, additional questions were raised about whether repetitive head impacts with magnitudes below those typically associated with diagnosed concussions may change concussive tolerance or eventually manifest as structural, cognitive and neuropsychiatric changes typically associated with the onset of concussion.^2^,^31^,^34^ The vast majority of head impacts in sports are of relatively low magnitude and usually not associated with clinically-identifiable concussion.^15^ For example, Rowson and Duma recorded approximately 63,000 head impacts using the HIT System with only 37 diagnosed concussions.^45^ On an individual basis, athletes were shown to sustain a high number of repetitive head impacts through routine participation in football. Broglio and colleagues recorded over 32,000 head impacts in 42 high school football athletes during a 15-week season, resulting in an average of 774 recorded head impacts per player.^11^ The number of head impacts per player varied by position, with linemen sustaining the highest number and quarterbacks receiving the fewest. Focusing on effects of head impact exposure in non-concussed athletes, Talavage and colleagues identified measurable changes in neurocognitive performance and functional magnetic resonance imaging (fMRI) metrics associated with routine participation in high school football.^51^ The magnitudes of those changes were correlated to the number of repetitive head impacts.^8^ Similarly, McAllister and colleagues reported that changes in white matter diffusivity in college football athletes were correlated with measures of head impact exposure.^35^ Other studies reported similar findings.^2^,^16^,^30^,^34^

Despite the focus on biomechanics of concussive events and correlation of repetitive head impacts to neuropsychological and magnetic resonance imaging changes in the brain, limited effort was made to correlate concussion onset with repetitive head impact exposure. Findings from studies referenced above imply that repetitive head impacts likely decrease biomechanical tolerance for concussion resulting in the athlete being more susceptible to injury from lower magnitude impacts. In 2007, Guskiewicz and colleagues hypothesized a role for repetitive head impact exposure due to considerable variability in concussion biomechanics for their sample of 13 concussed college football athletes, with some athletes experiencing concussion onset following low magnitude impacts.^25^ Beckwith and colleagues provided data to support this hypothesis by demonstrating that athletes with delayed concussion reporting had a significantly higher number of head impacts on the day of injury and within seven days of injury.^4^,^5^ These findings have contributed to a line of thought that recent and/or lifelong repetitive head impact exposure may be a contributing factor to decreased concussive tolerance and concussion onset.

## Methods

This study was conducted to provide an analysis of the possible role for repetitive head impact exposure in the biomechanical onset of concussion in college football athletes. Head impact exposure of concussed National Collegiate Athletic Association (NCAA) Division 1 FBS football athletes was compared to each injured athlete’s controls, matched for both team and playing position, to determine if injured athletes had a higher volume or severity of head impacts than athletes that were not concussed. Head impact exposure for concussed athletes was compared to their position- and team-matched controls for a direct comparison between athletes participating in similar practice activities over the same time period.

### Population and Data Collection

Data included in this investigation are a subset of the NCAA-DoD Grand Alliance Concussion Assessment, Research, and Education (CARE) Consortium. Methods were described elsewhere^12^ and the study protocol was approved by the Institutional Review Board (IRB) at the Medical College of Wisconsin (MCW), with local sites utilizing a reliance agreement with the MCW IRB. Varsity college football athletes from six NCAA Division I FBS programs were consented and enrolled. Those programs included the United States Air Force Academy, the United States Military Academy at West Point, the University of California Los Angeles (UCLA), the University of North Carolina at Chapel Hill (UNC), the University of Wisconsin, and Virginia Tech (VT). Athletes from UCLA, UNC, and VT participated in 2015 and athletes from all six institutions participated in 2016 and 2017. Each athlete was equipped with the helmet-based Head Impact Telemetry (HIT) System^18^ (Riddell SRS, Riddell, Rosemont, IL, USA), which measures head linear accelerations using six accelerometers inside the football helmet and computes peak component and resultant linear and rotational accelerations. HIT System encoders were included in Riddell Speed and SpeedFlex helmets. Data acquisition triggered any time a single accelerometer exceeded a 9.6-g threshold. Only impacts with peak resultant linear acceleration greater than 10 g were included for analysis. Accelerations under 10 g can be associated with non-impact dynamic movements in the athlete. Head impact data were wirelessly transmitted to a laptop computer on the sidelines, and included peak resultant linear and rotational accelerations. Data were then stored in the Riddell cloud storage. The study team was provided access to the HIT System data in the cloud or by direct transfer from the local site* via* a secure ftp server. Personal identifying information was removed from the data by assigning a study specific number to each player.

Head impact data were recorded for all practice, scrimmage and game activities during the 2015, 2016, and 2017 football seasons, including spring practice, preseason training camp, and regular season practice and games. Local athletic medicine staff and study coordinators were responsible for assignment and maintenance of the HIT System, including placement in the helmets, charging, data offload and notification of the study team of any defective systems.

Concussions were identified and diagnosed by team medical staff, according to a standardized protocol outlined by the CARE Consortium.^12^ Specifically, concussion was defined according the consensus definition from the U.S. Department of Defense (DoD) evidence-based guidelines initiative, which closely parallels the AAN definition.^14^ Concussed athletes were entered into the CARE concussion protocol, consisting of repeated clinical assessments with advanced imaging and blood biomarker analysis. Local study team members recorded detailed data on the injury date and time, type of activity (practice, scrimmage or game), type of play and direction of head impact. Following notification of the injury, the study team secured the HIT System and video data for the date of injury and participated in a detailed analysis to identify the head impact associated with concussion onset. The analysis included at least two team members of the CARE Advanced Research Core (ARC) Head Impact Measurement Core and accounted for information in the concussion report, as well as head impact and video data from the injury date.

### Analysis of Repetitive Head Impact Exposure

Repetitive head impact exposure was assessed both on the injury date and from the initiation of activities for the season in which the player was injured (spring or fall of 2015, 2016, or 2017) through the injury date. Multiple athletes sustained concussion during March and April. Therefore, repetitive head impact exposure was quantified during those times. However, spring football was treated as a separate ‘season’ from fall football because activities occurred during time periods that were somewhat distant from fall with extended periods without contact activities between the fall and spring seasons. Therefore, the effect of repetitive head impacts for the onset of concussion was less immediate. Accordingly, six distinct ‘seasons’ were analyzed consisting of March–April and August-December 2015, 2016, and 2017.

Head impact exposure was quantified for each athlete using the number of recorded head impacts and a *cumulative injury risk metric* (i.e., risk weighted exposure^52^) that was calculated based on the cumulative severity of recorded head impacts. Both metrics were quantified on the day of injury and for the season up to and including the injury date. The risk weighted exposure metric (RWE) assigned a risk of injury to each head impact based on the peak magnitude of linear ($\ddot{x}$) and rotational ($\ddot{\theta}$) acceleration, according to the concussion probability equation developed by Rowson and Duma,^45^ shown below in Eq. (1). The RWE was then calculated as the sum of the risk associated with all head impacts over the given period (i.e., day of injury or season through the injury date).

1 $${\text{RWE}} = \mathop \sum \nolimits \frac{1}{{1 + e^{{ - ( - 10.2 + 0.0433*\ddot{x} + 0.000873*\ddot{\theta}- 0.000000920*\ddot{x}*\ddot{\theta})}}}}$$

### Selection of Controls

Selection of control athletes was focused on the identification of non-concussed athletes that were exposed to the same practice and game conditions as the concussed athletes, acknowledging that positional hierarchy (i.e., starters vs. backups) was not actively tracked or updated by the study. Accordingly, head impact exposure for each concussed player was compared to their position- and team-matched controls. In other words, head impact exposure for an injured defensive back at University 1 was compared to other defensive backs at the same University. While this limited the total number of controls for each injured athlete, it provided a very specific comparison between athletes that had very similar contact activity profiles. Practice days, times, and activities vary between programs and position groups, and this analysis eliminates much of that variability. Athletes were grouped into seven primary position groups including defensive backs, linebackers, defensive linemen, offensive linemen, receivers, offensive backs, and quarterbacks. Defensive backs included cornerbacks and safeties. Receivers included wide receivers and tight ends. Offensive backs included running backs and fullbacks. Kickers/punters were not included in the analysis as there were no concussions sustained for athletes in those position groups. Each concussed athlete was then matched to all enrolled athletes in his position group that also played for his team. However, controls that participated in greater than 33% more or fewer days of contact football activities from the start of the ‘season’ through the injury date were excluded from the analysis since the objective of these comparisons was to compare head impact exposure between athletes that had similar contact activity participation. A majority of injured athletes were matched to more than 1 control participant using this method, allowing us to analyze a more meaningful distribution of non-injured athletes.

In addition to the team- and position-specific comparison, head impact exposure for concussed athletes was also compared to the entire uninjured control population. The number of head impacts and RWE was calculated for each day of participation for all controls. Those metrics for each injured athlete on the day of injury were compared to the distribution from the uninjured population. Specific attention was paid to concussed athletes that sustained a number of impacts or RWE on the day of injury that exceeded the 75th and 90th percentile for the non-concussed population.

### Statistical Analysis

Two separate statistical analyses were performed to discover factors differentiating concussed athletes from their matched controls. Both analyses took into account the “k controls” to “1 concussed” athlete matching. In the first approach, a Rank Score was calculated for each concussed athlete in his respective matched-control group according to Eq. (2) below.

2 $${\text{Rank Score }} = \, \left( {{\text{concussed athlete'}}{\text{s rank }}{-} \, 0.5} \right)/\left( {k + 1} \right)$$

where “*k*” equals the number of matched controls. The formula for Rank Score ensured that the average expected Rank Score was always equal to 0.5 irrespective of the number of matched controls. Rank Score was calculated for each concussed athlete and the average score was tested vs. the expected score of 0.5, which indicated no difference between the concussed and control groups.

In the second analysis, the actual scores of the considered factors were treated using a mixed effects approach which took into account matching* via* a group-specific (concussed athlete and his respective controls) random intercept effect. Each factor was considered as an outcome and the group membership (concussed vs. control) as a predictor. Factors tested in this analysis included the number of head impacts, RWE, and median linear and rotational head accelerations for all head impacts over the period of interest. The group membership effect was tested for a significant difference from zero.

Results of these analyses were statistically significant when the *p* value was less than 0.05 and as marginally significant when the p-value was between 0.05 and 0.10. A third analysis was performed for RWE measures, wherein the number of contact activities that the injured athlete participated in was iteratively limited to increasing numbers and athletes participating in fewer contact activities than the threshold were removed from the analysis. The purpose of this analysis was to identify a possible threshold for participation for which cumulative head impact exposure would have an increased influence on the mechanism of concussion.

## Results

**Figure 1:**
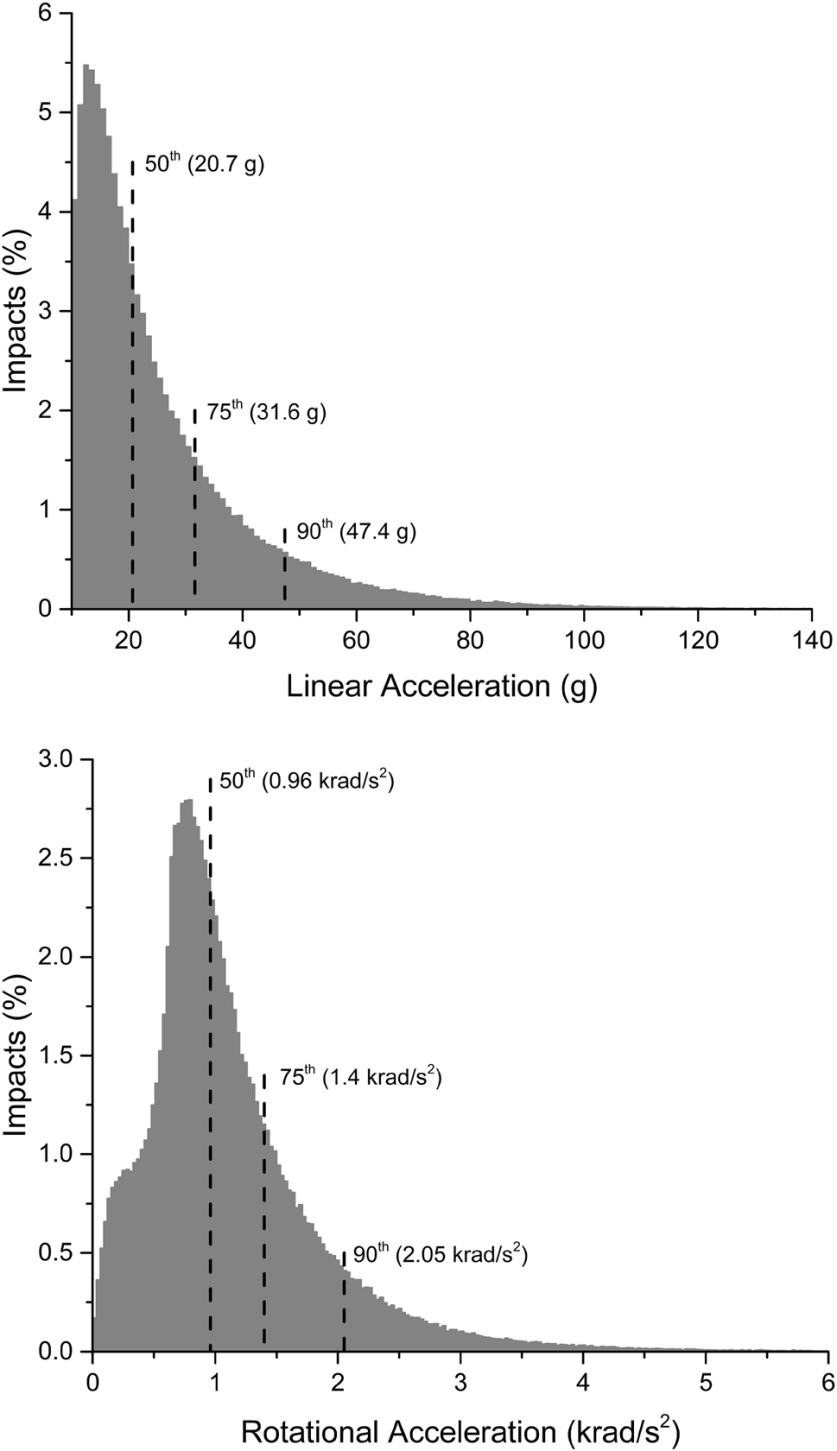
Histogram representations of the distribution of peak linear (top) and rotational (bottom) acceleration for all head impacts recorded in the 454 non-concussed athletes enrolled in this study.

Five hundred eleven (511) college football athletes from six NCAA Division 1 FBS college football programs were consented and enrolled. Head impacts were recorded using the HIT System for all football activities during the spring and fall 2015, 2016, and 2017 seasons. A total of 424,059 head impacts were recorded across 484 unique dates and 34,267 athlete exposures. The distribution of peak resultant linear and rotational acceleration for head impacts sustained by all non-concussed athletes is presented in Fig. 1.

Fifty athletes sustained concussion while wearing the HIT System. Nine concussions occurred during the 2015 calendar year, 17 concussions during the 2016 calendar year, and 24 concussions during the 2017 calendar year. Interestingly, a majority of concussions occurred early in the season. Twenty-four of the 50 concussions (48%) occurred during the month of August. Five concussions (10%) occurred during September, ten (20%) during October, three (6%) during November, and two (4%) during December. An additional six concussions (12%) occurred during Spring football practices (March/April).

Concussions were sustained by athletes in all primary playing position categories. Offensive linemen (*n* = 9), defensive backs (*n* = 9), and linebackers (*n* = 9) were most affected. Running backs (*n* = 7), wide receivers/tight ends (*n* = 7), and defensive linemen (*n* = 7) formed the second highest affected group. Finally, quarterbacks (*n* = 2) were affected least often. No enrolled kickers/punters sustained concussion. When separating concussed athletes by length of participation for the season prior to concussion, some differences by playing position were evident. For example, all seven defensive linemen were injured after participating for ten or more days of contact activities. In contrast, seven of the nine injured defensive backs and five of seven wide receivers/tight ends were injured after participating in fewer than ten days of contact activities.

The concussive impact was identified for 50 concussions. Ninety-one percent of concussive impacts identified using the HIT System matched the direction of head impact identified on video analysis. Recorded head impacts that did not match the video were shown to occur to the lateral side of the head (right or left) on video and were recorded as occurring to the back of the head in the HIT System. Mean linear accelerations for the 50 impacts were 71 ± 30 g’s (median: 65 g) and mean rotational accelerations were 3379 ± 1775 rad/s^2^ (median: 3050 rad/s^2^). Head impacts for 28 of the 50 concussions (56%) were associated with a probability risk of less than 1% according to a previously published concussion risk relationship.^45^ In the case of these low magnitude concussive impacts, no other head impacts were recorded on the injury date with associated risk greater than 1%. Head impacts for 10 of the 50 concussions (20%) were associated with a risk of greater than 10% (greatest risk: 80%). Head impacts for the remaining twelve concussions were associated with a risk between 1 and 10%. In the event of low magnitude impacts, queries were submitted to Riddell to ensure that higher magnitude head impacts had not been filtered out around the time of the concussive impact. In sum, non-concussed athletes sustained 4589 head impacts with greater linear and rotational acceleration than the mean accelerations for concussed athletes, and 249,160 head impacts with greater linear and rotational acceleration than the lowest magnitude concussive impact. These findings highlight the difficulty in predicting concussion in contact sports based on the magnitude of a single head impact. While the single impact mechanism likely contributed to concussion in some cases, other concussions were clearly the result of factors other than the head impact sustained immediately prior to injury.

### Comparison to Team- and Position-Matched Controls

Head impact exposure data for each of the 50 concussed athletes and the associated team- and position-matched controls are presented in Tables 1, 2, 3, and 4. Concussed athletes matched to an average of 4.4 ± 2.6 matched control athletes for the injury date (range 0–10) and 3.8 ± 2.5 matched controls for the season to the injury date (range 0–10). Forty-five concussed athletes matched to two or more controls for the analysis of head impact exposure on the injury date (Tables 1, 2) and 42 concussed athletes matched to two or more controls for the analysis of head impact exposure for the season to the injury date (Tables 3, 4). Two athletes had no matched controls for either analysis period (day/season of concussion), another two athletes had no matched controls only for the day of concussion analysis, and two separate athletes had no matched controls only for the season of concussion analysis.

**Table 1:**
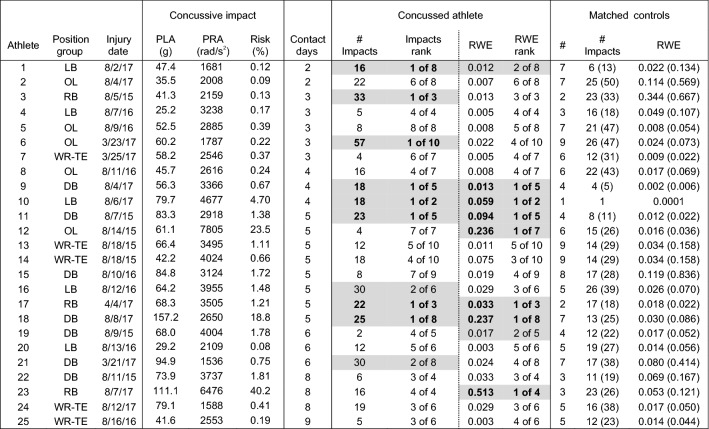
Peak linear (PLA) and rotational (PRA) accelerations, and associated risk, for the concussive impact, and number of impacts and risk weighted exposure (RWE) on the **date of concussion** for athletes participating in less than 10 days of contact activities prior to concussion.

Concussive impacts with video verification of impact location are scaled for certainty correction according to Ref. 48. Athletes ranked 1 or 2 in their matched control group for number of impacts or RWE are highlighted in gray. Number of matched controls (#) are presented, as well as mean(maximum) for the non-concussed controls for number of impacts (# impacts) and RWE

**Table 2:**
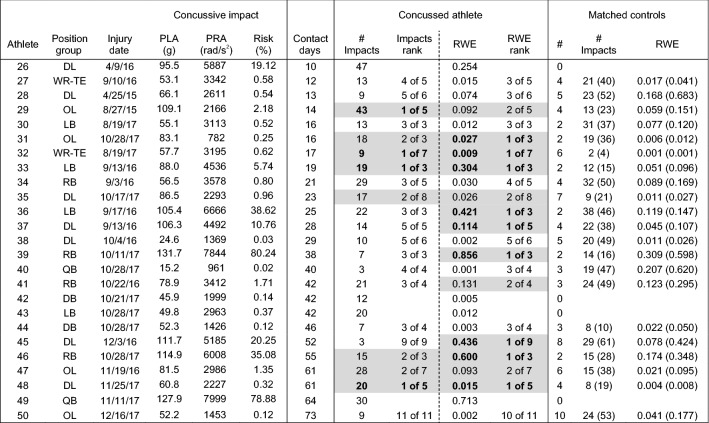
Peak linear (PLA) and rotational (PRA) accelerations, and associated risk, for the concussive impact, and number of impacts and risk weighted exposure (RWE) on the **date of concussion** for athletes participating in more than 10 days of contact activities prior to concussion.

Concussive impacts with video verification of impact location are scaled for certainty correction according to Ref. 48. Athletes ranked 1 or 2 in their matched control group for number of impacts or RWE are highlighted in gray. Number of matched controls (#) are presented, as well as mean(maximum) for the non-concussed controls for number of impacts (# impacts) and RWE

### Comparison to Team- and Position-Matched Controls: Day of Injury

Twelve concussed athletes (27%) sustained the highest number of head impacts for their matched control group on the date of injury (Tables 1, 2). Another six athletes had the second highest number of head impacts for their matched control group. One athlete had 47 head impacts on the injury date, the second highest for all concussed athletes, but had no matched controls. With regard to the cumulative risk metric (RWE), sixteen athletes (36%) had the highest value for their matched control group on the injury date. Another six athletes had the second highest RWE for their matched control group. One athlete had a RWE of 0.713, the third highest for all concussed or matched control athletes, but had no matched controls. One concussed athlete had a greater number of head impacts and greater RWE on the injury date than his one matched control.

Of the athletes that had at least one matched control on the injury date, 20 (43%) had the highest number of head impacts for their matched control group, the highest RWE for their matched control group, or both (*n* = 8). Twenty-six concussed athletes (57%) were ranked 1 or 2 of their matched control group for either the number of impacts on the injury date, RWE on the injury date, or both (n = 14). In general, the number of athletes with high exposure on the injury date was not different between athletes that participated in fewer than 10 sessions prior to concussion (Table 1) and athletes that participated in more than 10 sessions prior to concussion (Table 2).

### Comparison to Team- and Position-Matched Controls: Season of Injury

Comparison of head impact exposure for concussed athletes to team- and position-matched controls for the entire season up to and including the injury date revealed somewhat stronger trends. Eleven concussed athletes with two or more controls (26%) sustained the highest number of head impacts for their matched control group for the season up to and including the injury date (Tables 3, 4). Another 17 athletes (41%) had the second highest number of head impacts for their matched control group. Accordingly, 67% of concussed athletes were Rank 1 or 2 in their matched control group for the number of head impacts for the season up to the injury date, compared to 40% for the number of head impacts on the injury date.

**Table 3:**
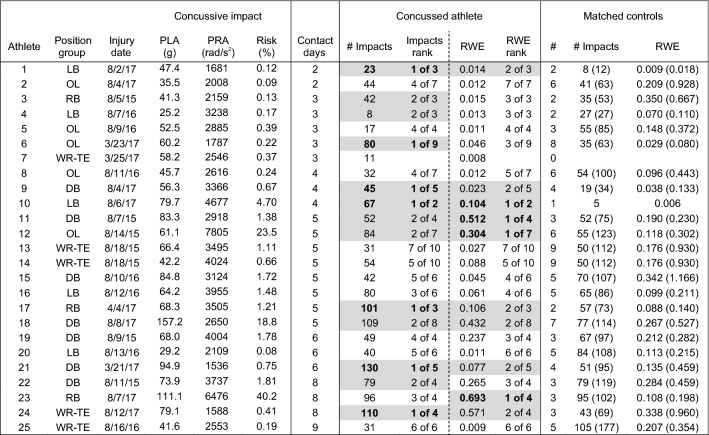
Peak linear (PLA) and rotational (PRA) accelerations, and associated risk, for the concussive impact, and number of impacts and risk weighted exposure (RWE) for the **season** up to and including the date of concussion for athletes participating in less than 10 days of contact activities prior to concussion.

Concussive impacts with video verification of impact location are scaled for certainty correction according to Ref. 48. Athletes ranked 1 or 2 in their matched control group for number of impacts or RWE are highlighted in gray with Rank 1 in bold. Number of matched controls (#) are presented, as well as mean(maximum) for the non-concussed controls for number of impacts (# impacts) and RWE

**Table 4:**
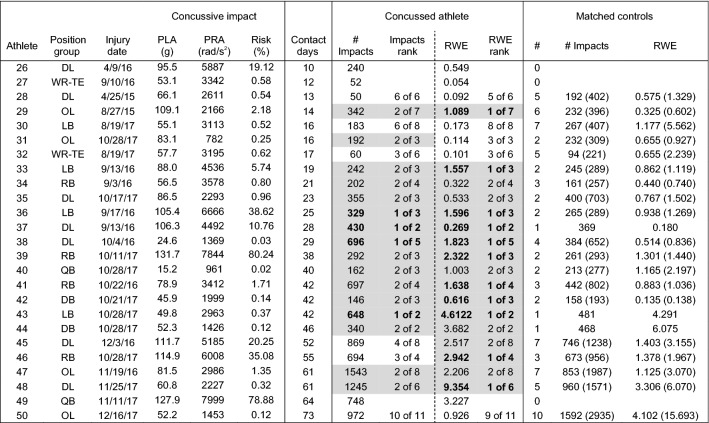
Peak linear (PLA) and rotational (PRA) accelerations, and associated risk, for the concussive impact, and number of impacts and risk weighted exposure (RWE) for the **season** up to and including the date of concussion for athletes participating in less than 10 days of contact activities prior to concussion.

Concussive impacts with video verification of impact location are scaled for certainty correction according to Ref. 48. Athletes ranked 1 or 2 in their matched control group for number of impacts or RWE are highlighted in gray with Rank 1 in bold. Number of matched controls (#) are presented, as well as mean(maximum) for the non-concussed controls for number of impacts (# impacts) and RWE

With regard to the cumulative risk metric, fifteen athletes with at least one matched control (33%) had the highest RWE for their matched control group for the season up to and including the injury date. Another eleven athletes had the second highest RWE for their matched control group. Therefore, 57% of concussed athletes were Rank 1 or 2 in their matched control group for RWE for the season up to the injury date, compared to 48% for the date of injury.

Of the athletes that had at least one matched control for the season up to and including the injury date, 21 (46%) had the highest number of head impacts for their matched control group, the highest RWE for their matched control group, or both (*n* = 5). Thirty-one concussed athletes (67%) were ranked 1 or 2 of their matched control group for either the number of impacts, RWE for the season up to and including the injury date, or both (*n* = 23), compared to 57% for the date of injury.

Predictably, the influence of repetitive head impact exposure in concussion onset was stronger for athletes that participated in a greater number of contact sessions. For example, the number of concussed athletes with the highest RWE for their matched control group was greater for athletes that participated in more than 10 contact sessions (Table 4; *n* = 11, 50%) than athletes that participated in fewer than 10 contact sessions (Table 3; *n* = 4, 17%). Likewise, 17 unique concussed athletes (77%) that participated in more than 10 contact sessions were Rank 1 or 2 for their matched control group for number of impacts for the season to the injury date, RWE for the season to the injury date, or both (*n* = 14), compared to 14 (58%) concussed athletes that participated in fewer than 10 contact sessions. Likewise, athletes participating in more than 10 days of contact activities had a significantly lower (*p* < 0.05) cumulative Rank Score for RWE (average 0.286 ± 0.234) compared to athletes that participated in fewer than 10 days of contact activities (average: 0.429 ± 0.231), indicating a rank within their matched control group closer to 1.

### Statistical Analysis of Matched Control Groups

Trends highlighted above were supported by statistical analyses. In the first approach, the Rank Score was calculated for each considered factor and tested vs. the expected average score equal to 0.5. Including all 46 concussed athletes with matched controls, the Rank Score for RWE was found to be marginally statistically significantly lower for the concussed group (*p* value = 0.090), indicting a higher RWE and a Rank closer to 1 for the concussed athletes. That difference became statistically significant as athletes that participated in fewer days of contact activities were removed from the analysis. When limiting the comparison to only athletes that participated in three or more days of contact activities, concussed athletes had a Rank Score for RWE that was significantly lower (*p* = 0.014) than the matched control group. That difference remained statistically significant as the analysis was limited to athletes with even longer contact participation periods. For example, athletes that participated in five or more (*p* = 0.035) and ten or more (*p* = 0.014) days of contact activities also had a Rank Score for RWE that was significantly lower for concussed athletes than their matched controls. These findings indicate that concussed athletes had higher exposure than their matched controls for the season leading up to the injury date. However, contrary to the findings for RWE, this statistically-based Rank Score analysis did not reveal significant differences between concussed and matched control groups based on the number of head impacts for the season leading up to the injury date.

The second statistical analysis was a groupwise comparison of absolute values for RWE, number of impacts, and magnitudes of linear and rotational acceleration on the injury date and for the season up to the injury date. For the injury date, number of impacts, RWE, and median peak linear acceleration were not significantly different between concussed and matched control groups. However, median peak rotational acceleration was significantly greater in the concussed group (*p* = 0.041), which was likely attributable to the higher magnitude concussive impacts that some of the concussed athletes sustained. Focusing on the season up to the injury date, RWE, number of impacts, and median peak linear and rotational accelerations were also not significantly different between concussed and matched control groups (*p* > 0.10). However, as the minimum participation was increased to 5 days, RWE was marginally statistically significantly greater for the concussed group (*p* = 0.084). This finding supports the Rank analysis highlighted above by demonstrating stronger exposure-based trends for athletes that participated in contact activities for an increased number of days and highlighting the influence of RWE as a possible predictor for concussion onset.

### Comparison to the Entire Uninjured Population

**Figure 2:**
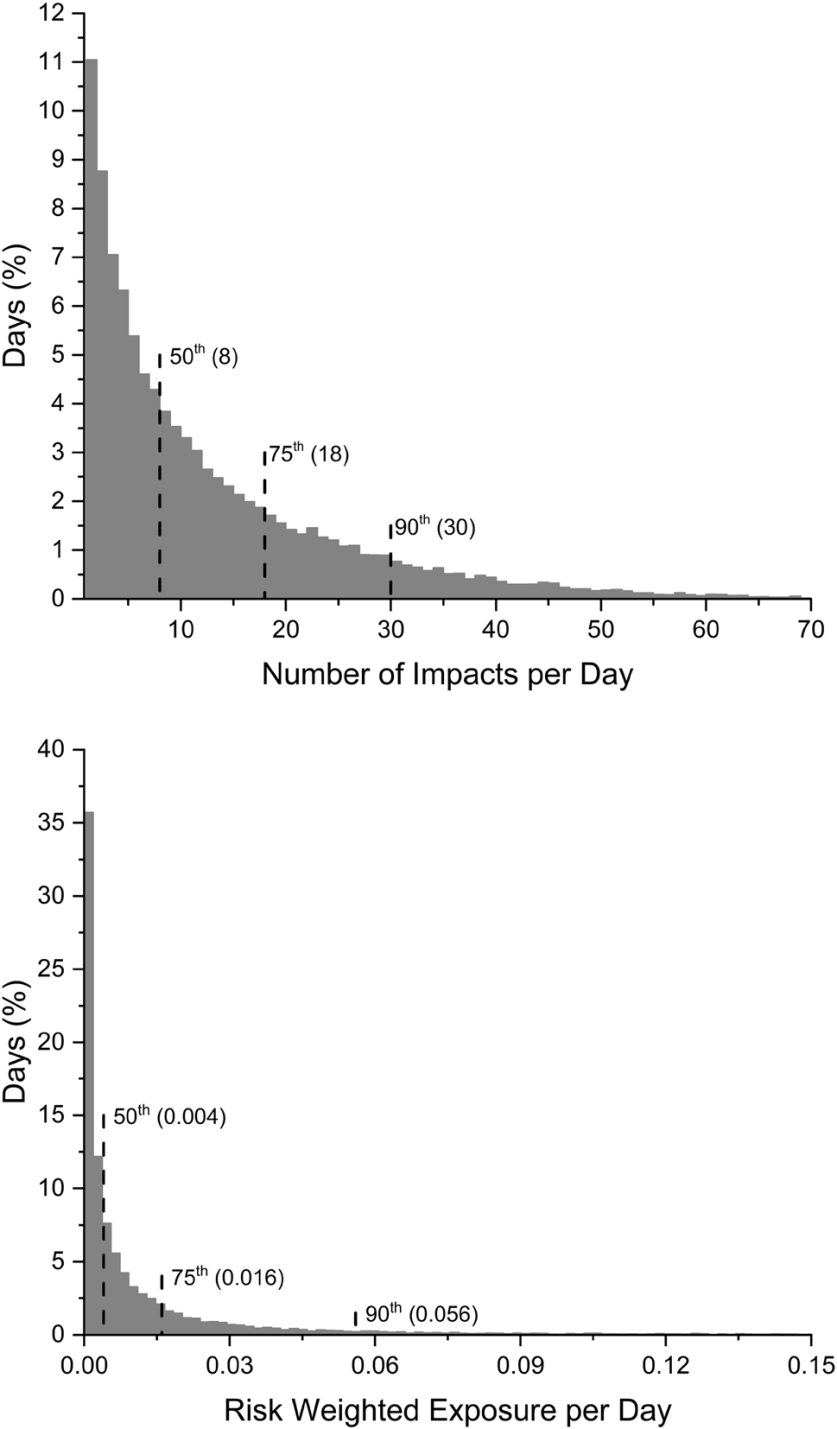
Histograms of distribution of number of head impacts per day and risk weighted exposure (RWE) per day. Data are presented for the non-concussed population (*n* = 454). 50th, 75th, and 90th percentile values are presented in parentheses.

Repetitive head impact exposure in concussed athletes can also be compared to the distribution of head impact exposure from the 454 uninjured athletes. The distributions of the number of head impacts per day and RWE per day for the uninjured population is presented in Fig. 2. The median, and 75th and 90th percentile for the number of head impacts per day was 8, 18, and 30 head impacts. Likewise, the median, and 75th and 90th percentile for RWE per day was 0.004, 0.016 and 0.056. Focusing on head impact exposure for the day of concussion, 23 injured athletes (46%) had equal to or more than the 75th percentile for the number of head impacts per day for the entire uninjured control population. Seven injured athletes had more than the 90th percentile. Likewise, 29 of 50 concussed athletes had RWE greater than the 75th percentile for the uninjured population on the injury date. Therefore, 58% of injured athletes had RWE greater than 0.016 on the date of injury compared to only 25% of the uninjured population for any given day of football activities. Eighteen injured athletes had more than the 90th percentile, indicating that 36% of concussed athletes had RWE greater than 0.056 on the day of injury compared to only 10% of the uninjured population for any given day of football activities.

The average magnitude of head impacts can also be compared between concussed and non-concussed populations. The distribution of head impact severity for all head impacts sustained by non-concussed athletes is shown in Fig. 1. The median, 75^th^, and 90^th^ percentiles for peak linear acceleration of all head impacts sustained by concussed athletes was 21.1, 32.6, and 49.8 g’s. Likewise, the 50th, 75th, and 90th percentiles for peak rotational acceleration of all head impacts sustained by concussed athletes was 972, 1463, and 2122 rad/s^2^. Accordingly, the distribution of head impact magnitudes between concussed and non-concussed athletes was remarkably similar, varying by 5% or less for average and high magnitude impacts.

## Discussion

The present analysis identified the role of repetitive head impact exposure as a moderator for concussive injury in some college football athletes. Overall, 72% of concussed athletes in this study had evidence of elevated head impact exposure either on the injury date or for the season up to and including the injury date when compared to their team- and position-matched controls. That evidence consisted of injured athletes sustaining the highest number of head impacts and/or having the highest RWE over the period of study. Risk weighted exposure is particularly interesting, as it is an assessment of both the number and severity of head impacts over the given time period. Stronger association of an elevated RWE with the onset of concussion implies a role for the number and severity of head impacts as moderating factors in the eventual onset of concussion. Ten athletes (22%) had the highest RWE for their matched control group both on the injury date and for the season up to and including the injury date. However, at least in the case of season-long exposure, each concussed player that had the highest RWE of their matched control group, with only two exceptions (13/15), also had the highest or second highest number of head impacts over the period of analysis. This implies a stronger role for the number of repetitive head impacts for injured athletes along with a relatively consistent head impact profile in terms of the magnitude of linear and rotational accelerations for injured and control athletes. Results of this study demonstrated consistency with regard to the severity of repetitive head impact exposure between injured athletes and controls, with acceleration magnitudes of routine (50% percentile) and higher magnitude (75th and 90th percentile) head impacts varying by 5% or less between the two groups.

These findings support the increasing trend toward policies intended to limit head impact exposure during football practice activities. For example, the Ivy League NCAA football conference moved to eliminate tackling during all football practices in an effort to minimize head impact exposure outside of games.^6^ That type of decision was influenced by research that identified a correlation between an elevated number of head impacts and changes in cognitive assessments and/or neurophysiology,^2^,^7^,^8^,^35^,^51^ although that finding has not had unanimous support in literature.^26^,^38^ The present study provides evidence that the number and/or severity of repetitive head impacts may play a role in concussion onset. This is further supported by the timing of concussions in this study, wherein 48% of all concussions occurred during the month of August and 60% of concussions occurred in August or during spring practices. That timing is significant as it indicates that concussions occurred prior to the initiation of game activities and during a time period where athletes may experience increased head impact exposure due to pre-season preparation activities and two-a-day practices. However, it should be noted that two-a-day practices during the preseason were eliminated by the NCAA in 2017 and the effect of this ruling on concussion rate during the preseason has not yet been reported. Interestingly, Swartz and colleagues^50^ demonstrated that helmetless tackling and blocking drills during practice can decrease the number of head impacts per athlete exposure by greater than 25%. This type of change in skills practice may provide some benefit over the long term by reducing overall head impact exposure and possibly decreasing concussion risk.

The finding of an increased number of concussions that occurred during the preseason may seem somewhat contrary to the repetitive exposure hypothesis presented here. That hypothesis would imply a greater risk for concussion associated with an increasing number of head impacts. Therefore, exposure would continue to increase throughout the season and athletes would be at greatest risk for concussive injury at the end of the season. A greater number of concussions occurring early in the season could have one of two possible explanations. The first explanation would be an exposure-based threshold that can be exceeded relatively early in the season for some athletes. Individual variability in single-impact concussive tolerance was recently identified,^47^ and may also hold true for exposure-based tolerance presented here. The second explanation would indicate some aspect of time/frequency in the development of increased concussion susceptibility associated with repetitive head impact exposure. This explanation may be more likely, given the recent finding that the timing between impacts (i.e., impact frequency) may influence injury risk.^10^ This could be the result of repetitive low-level injury and temporal neurometabolic dysfunction,^21^ with more frequent head impacts resulting in earlier disruption of the recovery process. However, these theories require further study and validation using finite element or animal models to characterize progressive effects of repetitive head impact exposure in relation to concussive risk.

### Selection of Controls

Findings from this study are in line with the work of Beckwith and colleagues, who recently reported an increased number and severity of head impacts for concussed athletes on the date of concussion compared to days without concussion.^4^,^15^ However, comparison of head impact exposure in concussed athletes to team- and position-matched controls represents a unique addition to this analysis, designed to focus the comparison on individual playing style, while removing effects of practice activities, coaching styles, and offensive/defensive schemes. This methodology was justified as previous studies demonstrated differences in football athlete head impact exposure by position,^11^,^15^ with linemen reported to sustain as many as two times the number of head impacts as defensive backs.^11^ The number of recorded head impacts was also shown to vary by team,^15^,^33^ with athletes from one team sustaining approximately 35% more head impacts per athlete exposure than another team. Therefore, the variability associated with those factors has been removed in the current analysis and differences evident in Tables 1, 2, 3 and 4 are more attributable to individual playing style. Accordingly, these results highlight considerable differences in head impact exposure that may subject a subset of athletes to higher risk of concussion based on their style of play. This finding would make a case for continuous head impact monitoring in contact sports. Conceivably, concussion risk can then be reduced by focusing on playing style adjustments in athletes with high head impact exposure to limit the number and severity of head impacts sustained throughout the season.

### Concussion Threshold

Consistent with previous studies incorporating similar data collection methodologies, the magnitude of head impacts resulting in concussion onset demonstrated a wide range for the 50 concussed athletes in this study. Concussion onset occurred following head impacts that had associated injury risks as low as 0.02%, indicating that concussion from a head impact of that severity would only be expected twice out of every 10,000 head impacts. The highest associated risk for any of the 50 concussive head impacts in this study was 80%. Mean values for linear and rotational acceleration were lower than earlier studies,^13^,^19^,^25^ although more recent studies reported magnitudes more in line with the current findings.^5^,^17^,^36^ This could be attributable to a number of factors, but may partially reflect an increasing recognition of concussion symptoms for on-field sports medicine personnel and athletes, which has occurred over the past several years. This increased awareness may have resulted in the identification or athlete reporting of lower severity concussions or those resulting from lower severity head impacts.

Perhaps more importantly, however, is consistency in terms of the variability in peak head accelerations associated with concussion onset. Studies highlighting concussion biomechanics generally reported some head impacts that were associated with a very low risk for concussion, while other head impacts were associated with very high risks. For example, over half of all concussions in this study (*n* = 28) were associated with head impacts that had biomechanics indicative of less than a 1% risk of injury according to a previously defined relationship,^45^ while the highest risk was 80%. McAllister and colleagues reported concussive head impacts with a similar range of injury risk.^36^ Even studies that reported higher mean accelerations than the current analysis demonstrated some concussive impacts associated with less than 1% risk.^25^ These findings highlight a possible need to re-think the way that head impact biomechanics may be used to assist in the identification of concussions in contact sport athletes. According to the studies referenced above, a majority of contact sport concussive impacts would not exceed a notable biomechanical threshold, and use of a lower threshold would lead to a very high number of false positive alerts. Present results would indicate that the study of head impact biomechanics may derive additional clinical utility from the analysis of head impact exposure in terms of the number and severity of repetitive head impacts. Accordingly, biomechanical assessment of contact sport athletes for the identification of possible concussions should include screening for both high magnitude single impacts as well as a cumulative analysis focused on high exposure.

### Risk Weighted Exposure for Concussion Risk Associated with Head Impact Exposure

The present analysis incorporated RWE that was used to weight the influence of the number and severity of repetitive head impacts in the eventual onset of concussion. The analysis provided interesting evidence that head impact exposure may influence concussion. However, the analysis highlighted a greater role for the number than the severity of head impacts. This may be partially attributable to the use of a metric focused on the risk of concussion for each individual head impact, which may have over weighted high magnitude impacts, while under weighting moderate severity impacts resulting from a steep rise in risk for head impacts in the 80–100 g^43^ and 5000–7000 rad/s^2^^46^ ranges. For example, the risk of a 100-g and 5000-rad/s^2^ head impact is twice that of an impact with biomechanics only 10% less and six times that of an impact with biomechanics 20% less. Given the likely incremental effect of repetitive head impact exposure, a more linear relationship may be reasonable for RWE. In addition, the frequency and direction of head impacts was not accounted for in this analysis. Direction of head accelerations has long been understood to influence concussion risk and severity,^20^,^29^ and recent studies have highlighted a possible role for head impact density in the onset of concussion.^10^ Continued collection and similar analyses to that presented here may provide more insight on a more accurate dose response metric.

Nonetheless, in this study season-long RWE demonstrated a robust relationship with concussion. For example, an argument could be made that a single high magnitude concussive head impact may artificially raise RWE, biasing results based solely on the most recent head impact. To address this issue, a secondary analysis was performed wherein the concussive impact was removed from the calculation of RWE for all concussed athletes to assess the influence of that impact in the overall analysis of exposure. Removal of the concussive impact changed the season rank (Tables 3, 4) for only 7 of the 50 concussed athletes. Concussive impacts for those seven were associated with injury risks of between 0.2 and 36.8% (mean: 14.3%). Five of the seven participated in 5 or fewer days of contact activities prior to concussion and only two of those five were previously ranked 1 or 2 for their matched control group. The other two athletes participated in 25 and 28 days of contact activities and their rank was decreased from 1 to 2 in both cases. This indicated that removal of the concussive impact had a marginal effect on this analysis of season-long RWE and the results of this study. However, as expected, removal of the concussive impact did have a much larger effect on the date of injury RWE analysis (Tables 1, 2), wherein the rank for 28 of the 50 concussed athletes was decreased within their matched control group.

### Influence of Head Impact Exposure

In the comparison of head impact exposure for concussed athletes to their team- and position-matched controls, 34 of the 48 concussed athletes with at least one matched control had evidence of significant head impact exposure on the day of injury or for the season leading up to injury. Of the remaining 14 athletes, another four had RWE on the day of injury that was greater than the 75th percentile for the entire non-concussed population. However, ten concussed athletes had no evidence of significant head impact exposure either on the date of concussion or for the season of concussion. Biomechanics of the head impacts leading to the onset of concussion in all of those athletes were also associated with less than a 1.25% risk of injury. For example, the average risk for head impacts resulting in concussion onset in those athletes was 0.36%, with eight of the ten resulting from head impacts less than 60 g and 3200 rad/s^2^. Therefore, although head impact exposure was likely a contributing factor in many of the concussions reported in this study, approximately 20% of the concussions had no strong biomechanical explanation. This clearly indicates that other factors beyond biomechanics can play a significant role in concussion onset. The reporting of post-concussion symptoms may be dependent on a number of factors including genetics, mental health history, current life stress, medical problems, chronic pain, depression, personality factors, and other psychosocial and environmental factors^53^ including motivation and excessive risk taking. Given the lack of strong biomechanical evidence for a portion of the concussed athletes in the present analysis, those factors may also play a role in either the onset or, more likely, the reporting of sports-related concussion.

### Limitations

A limitation of these analyses is that individual head impact exposure is known to vary between athletes. This was evident in data from the present analysis and others,^9^ and likely attributable, at least in part, to differences in playing exposure between athletes in terms of the number of games, practices, and repetitions for each athlete. Although this study controlled for the number of days that each athlete recorded head impacts and, to some degree, repetitions by selecting team- and position-matched controls, the number of repetitions may vary between athletes depending on whether the athlete is a starter or reserve and other factors including non-concussion injury status. As baseline assessments were only obtained at the time of athlete enrollment, starter/reserve status for concussed athletes in this study was not reliable. Another possible limitation of the current analysis is that undiagnosed concussions may have occurred, which were not accounted for in this dataset. In accordance with the study protocol and common parameters of concussion research, our results focus only on diagnosed concussive injuries. There is the possibility that other concussive injuries went unreported or undiagnosed, as indicated by prior studies.^1^

Similar to all types of instrumentation used to measure biomechanical signals, the Head Impact Telemetry System has an inherent level of inaccuracy and imprecision that can arise from helmet fit and usage, assumptions made during data processing, and the electrical instrumentation itself. Data collected using this system should be analyzed in light of these limitations. Laboratory validation of the HIT System has generally demonstrated positive results,^3^,^44^ although some studies have reported higher error values particularly for facemask impacts.^28^ More recently, Siegmund and colleagues performed an extensive laboratory comparison of HIT System data to accelerations measured using Hybrid III instrumentation and provided uncertainty equations based on helmet impact location.^48^ Accordingly, peak magnitudes of linear and rotational acceleration recorded using the HIT System can be corrected based on helmet impact location verified using video analysis. A limitation of this study is that only concussive impacts were verified using video analysis as our resources did not allow for video verification of all 424,059 impacts recorded during the study. Therefore, some level of inaccuracy may exist in this dataset, although the level of inaccuracy would be consistent with other studies using the HIT System Instrumentation.^5^,^10^,^26^,^33^,^47^ Additionally, since the inaccuracy would be expected to be consistent across athlete playing positions, based on the distribution of head impact locations, and since the current analysis demonstrated a stronger correlation with the number instead of the magnitude of head impacts, position matching in the current analysis is expected to somewhat limit these system inaccuracies.

## Conclusions

This analysis highlighted differences in repetitive head impact exposure between concussed athletes and controls that were matched for position and team. The purpose of the matching procedure was to control for opportunity for exposure in the same game and practice routines on the same days. Almost three quarters of the concussed athletes (72%) had the most or second most severe head impact exposure for their matched control group. Fifty-eight percent of concussed athletes had risk weighted exposure on the date of injury that was greater than the 75th percentile for the entire uninjured population (454 athletes). Therefore, this unique analysis provided some evidence for the role of repetitive head impact exposure in the onset of concussion for a cohort of concussed Division I college football athletes. While these trends require further validation, the clinical implication of these findings supports the contemporary trend of limiting head impact exposure for college football athletes during practice activities.
